# Mortality Statistics, Chicago

**Published:** 1875-06

**Authors:** 


***Chicago Mortality Report for April, 1875.*** Reported by Dr. BEN. C. MILLER, Sanitary Superintendent.

| | |
|---|---|
| Accident, crushed | 1 |
| " by fall | 3 |
| " by drowning | 3 |
| " by poison | 1 |
| " run over by buggy | 1 |
| " by railroad | 5 |
| Angina pectoris | 1 |
| Apoplexy | 4 |
| Asthma | 1 |
| Atelectasis pulmonum | 1 |
| Bowels, hæmorrhage of | 1 |
| Brain, congestion of | 5 |
| " disease of | 1 |
| " paralysis of | 1 |
| " inflammation of | 14 |
| " softening of | 1 |
| Bronchitis | 16 |
| " capillary | 11 |
| Cancer | 1 |
| " of abdomen | 1 |
| " of bowels | 1 |
| " of liver | 1 |
| " of stomach | 1 |
| " of uterus | 1 |
| Child birth | 1 |
| Cholera infantum | 14 |
| Consumption | 67 |
| Convulsions | 54 |
| " puerperal | 2 |
| Croup | 10 |
| " membranous | 3 |
| Cyanosis | 2 |
| Debility, general | 6 |
| Delirium tremens | 2 |
| Deficient development | 1 |
| Diarrhœa | 12 |
| " chronic | 2 |
| Diphtheria | 6 |
| Dropsy, general | 13 |
| " abdominal | 1 |
| Dysentery | 5 |
| Dyspepsia | 1 |
| Embolism | 1 |
| Endo-carditis | 4 |
| Enteritis | 13 |
| Entero-colitis | 1 |
| Epilepsy | 1 |
| Exposure | 2 |
| Erysipelas | 3 |
| Fever, congestive | 2 |
| " intermittent | 2 |
| " puerperal | 7 |
| " remittent | 4 |
| " scarlet | 17 |
| " typhoid | 10 |
| Fungus meduralis | 1 |
| Glands of throat, disease of | 1 |
| Gastritis | 6 |
| Gastro-enteritis | 7 |
| Hæmorrhage | 1 |
| Heart, disease of | 8 |
| " dropsy of | 2 |
| " fatty degeneration of | 1 |
| " organic disease of | 2 |
| " paralysis of | 1 |
| " valvular disease of | 4 |
| Hepatitis | 3 |
| Hydrocephalus | 6 |
| Inanition | 22 |
| Injury at birth | 1 |
| Intussusception | 1 |
| Indigestion | 2 |
| Icterus | 1 |
| Ilio colitis | 3 |
| Influenza | 1 |
| Kidneys, Bright's disease of | 2 |
| " inflammation of | 1 |
| Laryngitis | 1 |
| Liver, cirrhosis of | 2 |
| " disease of | 2 |
| " inflammation of | 1 |
| " hypertrophy of | 1 |
| Lungs, apoplexy of | 1 |
| " congestion of | 10 |
| " hæmorrhage of | 2 |
| " abscess of | 1 |
| " malformation of | 1 |
| Manslaughter | 1 |
| Metro peritonitis | 2 |
| Measles | 19 |
| Meningitis | 15 |
| " cerebro-spinal | 9 |
| " tubercular | 5 |
| Myelitis | 1 |
| Neurosis of pneumogastric | 1 |
| Ovaries, degeneration of | 1 |
| Œdema glottides | 1 |
| Old age | 16 |
| Paralysis | 3 |
| Pericarditis | 1 |
| Peritonitis | 4 |
| " puerperal | 2 |
| Pneumonia | 55 |
| " typhoid | 4 |
| Pleurisy | 2 |
| Rheumatism | 2 |
| " inflammatory | 1 |
| Scrofula | 3 |
| Septicæmia | 1 |
| Small-Pox | 3 |
| Spina bifida | 1 |
| Stomach, softening of | 2 |
| " hæmorrhage | 1 |
| Suicide, by cutting throat | 3 |

| | | | |
|---|---|---|---|
| Suicide by shooting | 2 | Uræmia | 3 |
| " by poison | 1 | Vitality, deficient | 1 |
| Syphilis | 2 | Whooping cough | 2 |
| Tabes mesenterica | 22 | | |
| Teething | 5 | Total | 644 |
| Tumor | 2 | | |

Premature births, 8; Still births, 69. Total, 77.

COMPARISON.

Deaths in April, 1875, 644; in March, 1875, 550. Increase, 94. Deaths in April, 1874, 486. Increase, 158.

AGES.

| | | | |
|---|---|---|---|
| Under one year | 248 | Twenty years to thirty | 59 |
| One year to two | 66 | Thirty " " forty | 56 |
| Two years to three | 30 | Forty " " fifty | 37 |
| Three " " four | 11 | Fifty " " sixty | 33 |
| Four " " five | 9 | Sixty " " seventy | 25 |
| Five " " ten | 23 | Seventy " " eighty | 22 |
| Ten " " twenty | 19 | Eighty " " ninety | 6 |
| | | Total | 644 |

| | | | | | |
|---|---|---|---|---|---|
| White | 636 | Males | 329 | Married | 192 |
| Colored | 8 | Females | 315 | Single | 452 |
| Total | 644 | Total | 644 | Total | 644 |

NATIVITIES.

| | | | | | |
|---|---|---|---|---|---|
| Africa | 1 | England | 11 | Scotland | 3 |
| Austria | 1 | Germany | 84 | Shetland Islands | 1 |
| Bohemia | 7 | Holland | 1 | Sweden | 9 |
| Canada | 9 | Ireland | 52 | Switzerland | 2 |
| Native—Chicago | 111 | Italy | 1 | Unknown | 8 |
| Foreign, " | 233 | Norway | 4 | | |
| U. States, other parts | 104 | Poland | 1 | Total | 644 |
| Denmark | 1 | | | | |

Deaths daily, 21½. Mean thermometer, 44°. Rain fall, 2.32 inches.

MORTALITY BY WARDS.

| Wards. | No. Deaths. | Pop. in 1874. | Percentage. | | Wards. | No. Deaths. | Pop. in 1874. | Percentage. | |
|---|---|---|---|---|---|---|---|---|---|
| 1 | 4 | 5,725 | one death in | 1,431 | 11 | 20 | 14,022 | one death in | 701 |
| 2 | 6 | 4,830 | " " | 805 | 12 | 19 | 16,792 | " " | 884 |
| 3 | 17 | 14,861 | " " | 874 | 13 | 29 | 17,892 | " " | 617 |
| 4 | 8 | 15,361 | " " | 1,920 | 14 | 29 | 16,720 | " " | 576 |
| 5 | 26 | 20,078 | " " | 722 | 15 | 95 | 45,545 | " " | 476 |
| 6 | 55 | 35,916 | " " | 653 | 16 | 41 | 21,922 | " " | 535 |
| 7 | 54 | 31,722 | " " | 587 | 17 | 21 | 20,777 | " " | 989 |
| 8 | 38 | 29,143 | " " | 767 | 18 | 39 | 21,392 | " " | 549 |
| 9 | 46 | 31,654 | " " | 688 | 19 | 7 | 4,677 | " " | 668 |
| 10 | 15 | 17,385 | " " | 1,159 | 20 | 10 | 8,995 | " " | 899 |

Ratio of deaths to population in 1874, one death in 614.

| | | | |
|---|---|---|---|
| No. deaths in Wards | 579 | Manslaughter | 1 |
| Accidents | 14 | Poisoned | 3 |
| Chicago River | 2 | St. Joseph's Hospital | 1 |
| County Hospital | 10 | Suicides | 6 |
| Foundlings' Home | 21 | Woman's Refuge | 1 |
| Hospital Alexian Bros. | 3 | | |
| Mercy Hospital | 3 | Total | 644 |

